# Supplementary material for: Understanding Antimicrobial Use Contexts in the Poultry Sector: Challenges for Small-Scale Layer Farms in Kenya
Source: Antibiotics (Basel). 2021 Jan 22;10(2):106. doi: 10.3390/antibiotics10020106 (PMC7911778; doi:10.3390/antibiotics10020106)
Supplement: Supplementary file 1 [file antibiotics-10-00106-s001.zip › antibiotics-1061982-supplementary/Supplement A.docx]

Supplement A: Qualitative Interview Guide

Interview guides

Themes and domains were developed during a workshop in Nairobi (August, 2018), where experts in antimicrobial resistance generated a list of themes/domains impacting antimicrobial use (AMU) and antimicrobial resistance (AMR) and probes to assess these themes/domains. See Table S1 for interview guides used during FGDs and IDIs with Kenyan layer farmers. See Table S2 for interview guides used for IDIs with animal health service professionals, including agrovets, livestock field officers, community animal health workers, and veterinarians.

**Table S1. Focus group discussion interview guide.** Themes/Domains were developed during a workshop in Nairobi (August 2018), where experts in antimicrobial resistance generated a list of themes/domains impacting antimicrobial use (AMU) and antimicrobial resistance (AMR) and probes to assess these themes/domains.

| **Themes/Domain** | **Topic and Probes *(in italics)*** |
| --- | --- |
| Economics / Masuala ya uchumi | 1. What livelihood activities does your community engage in?  - *What would you consider to be the five-priority livelihood activities based on contribution to household income? List from the most significant to the least* - *Can you estimate the percentage livestock income compared to other enterprise in a year?* - *During the year, when does the income from livestock profitable/ better compared to other enterprise?* - *What are the factors influence livestock process (per specie)? How do you ensure maximum profit?* |
| Management practices | 1. We would like to hear a bit about your animals that you keep.  - *What types do you keep and for what purposes?* - *How do you access resources (water & pasture), paying attention to seasonality?*   - *Sources of each resources and challenges faced including diseases*  1. Do you keep records of your farm management in general? If yes, how? If not why?  - *Probe about productivity, sales, purchases, movement of animals, treatments* - *Probe for the responsibilities of each focusing on gendered roles, in case of the Maasai- age set obligations, clan and household (boma) arrangements* |
| Environmental context / Mazingira | 1. Can you tell me how the environment you live in climate and weather conditions influence your farming practices? What impact do they have?  - *Probe for comparison on changes for the last five years* - *Do you experience interaction with wildlife while grazing, and how has this affect your farming?* - *Compared to other pastoral areas is the geographical position favourable or less favourable for livestock keeping?* - *In relation to population increase and livestock increase how have the farming practices been affected? With a focus of interaction with other livestock* - *Would you say living closely with other farmers or apart increase the chance of cross infections?* |
| Livestock Diseases | 1. Now we are going to talk about health problems that occur in our area, affecting livestock.  - *Can you tell me about the common diseases that your animals faced in the last year? List these per species.*   - *For each specie, lets rank from the most significant to the least.*   - *Where possible, discuss the top three, per season*   - *For the top three what were the common symptoms?*  1. For these diseases that you described above, can you tell me where they came from and how the animals were affected? 2. Health seeking practices  - *For each disease/ health condition what did you do?* - Probe on disease identification - *Probe on the treatment options taken* - *Where did you seek help and why?*  1. What is your preferred source of animal health information and why?  - *Probe on all possible angles, shops, friends, family, healers, drug peddlers, officials* - *What is your preferred source of medicines for treating livestock and why? Give examples of each* |
| Antimicrobial use | 1. In your day-to-day life do you talk of diseases caused by different kinds of parasites, such as bacteria’s and viruses? (probe on local generally disease causality understanding)  - Can you give examples of the different disease causative agents that you know?  1. In the process of identifying drugs for treating your livestock do you differentiate between antimicrobials and non-antimicrobials?  - How do you do that? - If not, how do you categorize drugs for treatment - *Do the options you take always work for you? Give experiences in the past year as far as you can remember* - Can you name the drugs and the possible diseases they can treat? Please explain how *and the exact measurements as per their understanding* - *Prompt specifically on the different places these are obtained* - *Ask for his opinion on these different sources focusing on issues of quality* - *Ask what strategies they use to decide from which source and why? Pay attention to elaborate on differentiating their own preferences and that of farmers* - *Prompt for experiences in carrying or storing practices by the farmers, and reasons for this*  1. Is there any difference between vaccines and other drugs including antimicrobials?  - *Can you give me examples of vaccines and when do you use them vaccine (ask for names with examples)* - *Where do you and farmers source antimicrobials for use in your community?*  1. In your farming practices, when do you normally administer antimicrobials? Probe for triggering actions such as    1. *Whether during prophylaxis, and why?*    2. *When an animal is sick with symptoms,*    3. *If the biosecurity and hygiene conditions are a course for concern* 2. *If time allows probe for reasons behind each use: whether all round costs, simply hygienic reasons, accessibility affecting quality etc* 3. How do you decide how much antibiotic (dose) to give to your animals, and for how long (treatment course) and how often?  - *Can you always get the kinds of antimicrobials you want to use? Why / why not?* |
| AMR | 1. Have you experience a situation whereby a drug is not working?  - *If yes, please give vivid examples and names of these drugs and what you intended to treat with?* - *How do you tell when a drug or the combination is not working?* - *When this happened what did you do?* - *Probe for all the options taken and why* - *If a combination of drugs was used before or after treatment failure ask for the names of the drugs and the amount of dose given to the animal?*  1. When this drug(s) do not work well as you explain before, how do you call this in your local terms or day-to-day farming practices?  - *Probe for any local or vernacular language, or if informed by the local service provider.* - *What do you think contributes/causes the drug not work properly to the extent you resort to other options? Give examples*  1. When you increase dosage of a drug what happens in the body of the animal?  - *To your understanding what is the meaning of drug volume or CC?* - *What drugs do you normally start with? What other drugs are given next. Give examples please as per disease* - *Can you explain how the following factors influence prescription and use of AMs? Costs of AMs,*   Withdrawal   1. After you treat your livestock and the treatment is not working  - *If you decide to slaughter and consume the meat when can you do that? What treatment is okay to use in the animal and slaughter the same day?*  1. *If you treat a livestock that you are milking what happens with the milk? (Please provide an example and the name of the drug)*  - *How do others in your community behave regarding withrdrawal? (discuss local context meanings and understandings of withdrawal?*  1. Do you normally talk among farmers (or animal health officers) about the relationship between drugs used in animals and whether they can get into humans?  - *If yes, what is the connection and how do you describe it in local lingo?* - *If they do get into humans what happens in the human body? What do you think is the outcome and why?* - *If no, do you think the drugs that are treated on livestock can get to humans through milk or meat? What do you think happens?*  1. If antimicrobials stopped working, what would that mean for you and your farm? |
| Summary and any other questions | *(Summarise the main points of the participant checking their agreement and asking if they have anything more to add).*  Are there any points you think I’ve missed, or any other issues you’d like to talk about relating to AMU and AMR? |

**Table 2.** Interview guide for in-depth interviews with animal health service professionals. .

| **Domain** | **Topic and Probes *(in italics)*** |
| --- | --- |
| Warm up | 1. I would like to hear a bit about your job. What does it entail? |
| Management practices | 1. What types of animals are kept by the community you serve?  - *How are they generally kept? Probe Free range or confines* - *Can you describe the common feeding practices to me?* - *Probe more on ranging systems, if confined how do they source the feeds, quality of feeds, problems with feeds, availability per season, storage practices*  1. Can you talk about the general hygiene practices of your farmers?  - *How often to they clean their pen?* - *What about disinfection practices? What disinfectants most commonly used?* - *What do you recommend could be the ideal hygiene practices in the context of the farmers you serve?*  1. Do the farmers you serve keep records?  - *Is it something that you encourage or its impractical to implement?* - *For those who keep records, what are the records about?* - *Probe about productivity, sales, purchases, movement of animals or treatment* - *What records of treatment are normally kept? Please explain* |
| General drug use in the local context | 1. Can you tell me the name of common drugs used to treat livestock in your area?  - *Probe for the names of diseases and the commonly used drugs* - *Probe about their own dispensing practices and later ask what farmers prefer* - *On whether treatment is categorized basing on different infections such as parasites, fungal or viral, bacteria* |
| Diseases | 1. What are the common livestock diseases in your area? 2. What are the top 5 most significant diseases affecting livestock you serve? Please name per specie 3. Can you rank the most significant to the least? Consider seasonality 4. How do livestock keepers approach you when their animals are sick?   Probe on stage of livestock disease |
| Understanding and categorization of antimicrobial in the local context | 1. Can you tell me the commonly used approaches and terms for describing antimicrobials in your work?  - *Probe for their own understanding of antimicrobials, if they do categorize basing on different infections*   - *Ask for specific drug names that are commonly available in the market* - *Probe for detail in how they inform the farmer that a particular drug is an antimicrobial, and if there is really a need to do so, and why?* - *Probe for the specific language, if any, used to communicate antimicrobials to farmers* |
| Sources and demand for antimicrobials | 1. Where do you and farmers source antimicrobials for use in the community?  - *Specifically on the different places these are obtained* - *Ask for his opinion on these different sources focusing on issues of quality* - *Ask what strategies they use to decide from which source and why? Pay attention to elaborate on differentiating their own preferences and that of farmers* - *Probe for experiences in carrying or storing practices by the farmers, and reasons for this* |
| Practices and experiences of AMU in community | 1. In your practice, when do you normally administer (or advice to farmers) antimicrobials? Probe for triggering actions such as  - Whether during prophylaxis, and why? - When an animal is sick with symptoms,   - If the biosecurity and hygiene conditions are a course for concern - Do farmers themselves have opinions on what antibiotics to use following your advice? |
| Understanding of AMR in the livestock value chain | 1. In your work have you come across a situation whereby an antibiotics have failed to work, say in the last year? If yes, probe about the following:  - *How do you call this phenomenon?* - *In this context have farmers encountered it? How do they refer to it?* - *Do you think AMR is caused by the animal being resistant to the drug? If yes or no, please explain* - *Do you think a micro- organism becoming resistant causes AMR? Explain both answers with examples* - *What else explains best AMR, what causes it, if the answers are different from above?* - *When treatment fails, what do people in the community do?*  1. What kind of activities that are performed by your farmers in the practice of rearing their livestock do contribute to the development of AMR? (Probe on the below statements if the respondent did not mention in his/her answers. Ask for vivid examples on each)  - *To what extent to you think your farmers contribute to AMR when they give drugs as per your instructions?* - *To what extent do farmers contribute to AMR if drug regimens are not completed, skipped, given doses that are lower than what you recommend* - *To what extent is AMR developed if farmers mix different types of antibioitics?* - *Given the general use of broad spectrum antibiotics, explain how its different uses do relate to AM.R Please give examples*  1. Do you encounter situations whereby farmers mix/ combine antibiotics in their animal feed?  - *Probe on how often this is a practice? Give examples* - *Probe if they consider such practices as contributing to the development of AMR* |
| Summary and any other questions | *(Summarise the main points of the participant checking their agreement and asking if they have anything more to add).*  Are there any points you think I’ve missed, or any other issues you’d like to talk about relating to AMU and AMR? |
