# Supplementary material for: Understanding Antimicrobial Use Contexts in the Poultry Sector: Challenges for Small-Scale Layer Farms in Kenya
Source: Antibiotics (Basel). 2021 Jan 22;10(2):106. doi: 10.3390/antibiotics10020106 (PMC7911778; doi:10.3390/antibiotics10020106)
Supplement: Supplementary file 1 [file antibiotics-10-00106-s001.zip › antibiotics-1061982-supplementary/Supplement B.pdf]

FINAL_KAP_SURVEY_GATUNDU

Study Introduction

[redacted]

We are interested in knowing how you manage your farm and we want to know this because we want to develop a program to help you improve the health of your animals

This survey will take between 1hr and 1.5hrs of your time

You do not have to agree to be interviewed and you can stop at any time choose and there will be no negative consequences for you

Anything you say during the discussion today will remain completely confidential: your name will not be used in any materials produced from this study nor any other information that could be used to identify me

You can request access to, moderations to, and/or deletion of my personal data.

Informed Consent

The study has been explained to me in a language that I comprehend. All the questions I had about the study have been answered.

I have been informed that it is my right to refuse to participate today and that if I choose to refuse I do not have to give a reason, and there will be no negative consequences for me.

I have been informed that anything I say during the discussion today will remain completely confidential: my name will not be used in any materials produced from this study nor any other information that could be used to identify me

I have been informed that I can request access to, moderations to, and/or deletion of my personal data.

I release FAO and its employees dealing with the use of my personal data as described above.

I agree to take part of this study

- ◯ Yes
- ◯ No

Please have them sign

1) Please Check Your Name

- ◯ [redacted]
- ◯ [redacted]
- ◯ [redacted]
- ◯ [redacted]
- ◯ [redacted]

2) Select Ward where interview will take place

- ◯ [redacted]
- ◯ [redacted]
- ◯ [redacted]
- ◯ [redacted]

3) Write name of the village

____

4) Household ID

____

5) What is your name?

____

6) What is your role in this farm?

Remember, try your best to interview owner

- ◯ farm owner
- ◯ spouse of farm owner
- ◯ son or daughter of farm owner
- ◯ farm manager
- ◯ employee that is not a manager

7) What year were you born?

Respondent

____

8) Record gender of respondent

- ◯ Male
- ◯ Female

9) What is gender of farm owner?

- ◯ Male
- ◯ Female

10) What year was farm owner born?

____

11) What is the owner's highest level of formal education?

- ◯ None
- ◯ Uncompleted Primary School
- ◯ Completed Primary Level
- ◯ Uncompleted Secondary Level
- ◯ Completed Secondary Level
- ◯ Uncompleted Tertiary Level
- ◯ Completed Tertiary Level
- ◯ Don't Know

12) What languages does the household read?

- ☐ English
- ☐ Kikuyu
- ☐ Kiswahili
- ☐ I don't read

13) What is your main occupation?

- ◯ Farmer
- ◯ Formal employment
- ◯ Informal employment
- ◯ Student
- ◯ Retired
- ◯ Other (specify)
- ◯ Other

Specify other.

____

14) How many people live on the farm?

____

15) How many employees does the farm have?

____

Now, I will give you a list of animals. I will ask you to tell me how many are at the house and whether they are in a pen or free-ranging

16) How many dairy cattle does the household have?

____

17) Are your dairy cattle?

- ◯ Free Range
- ◯ Confined
- ◯ Partially confined

18) How many beef cattle do you have?

____

19) Are your beef cattle?

- ◯ Free Range
- ◯ Confined
- ◯ Partially confined

20) How many sheep do you have?

____

21) Are your sheep?

- Free Range
- Confined
- Partially confined

22) How many goats do you have?

____

23) Are your goats?

- Free Range
- Confined
- Partially confined

24) How many pigs do you have?

____

25) Are your pigs?

- Free Range
- Confined
- Partially confined

26) How many dogs do you have?

____

27) How many rabbits do you have?

____

28) How many geese do you have?

____

29) Are your geese?

- Free Range
- Confined
- Partially confined

30) How many ducks do you have?

____

31) Are your ducks?

- ◯ Free Range
- ◯ Confined
- ◯ Partially confined

32) How many turkeys do you have?

____

33) Are your turkeys?

- ◯ Free Range
- ◯ Confined
- ◯ Partially confined

34) How many indigenous chickens do you have?

____

35) Are your indigenous chickens?

- ◯ Free Range
- ◯ Confined
- ◯ Partially confined

36) How many guinea_fowl do you have?

____

37) Are your guinea_fowl?

- ◯ Free Range
- ◯ Confined
- ◯ Partially confined

38) How many broilers do you have?

____

39) Are your broilers?

- ◯ Free Range
- ◯ Confined
- ◯ Partially confined

Now, I'd like to ask some questions specific to layers

What is the main reason you keep layers?

- ◯ Commercial (market oriented)
- ◯ Subsistence (own consumption and some income)
- ◯ Hobby

40) How are your layers kept?

- ☐ Cage System
- ☐ Deep Litter
- ☐ Semi-intensive

41) How many brooders (0-8 weeks) do you have?

____

42) How many pullets/growers (9-18 weeks) do you have?

____

43) How many layers (19-75 weeks) do you have?

____

44) How many spent layers (>75 weeks) do you have?

____

45) Do you keep multiple ages of layers at the same time?

- ◯ Yes
- ◯ No

46) How are multiple ages kept?

- ◯ I keep multiple ages in separate houses
- ◯ I keep multiple ages in the same house but seperated by A BARRIER
- ◯ I keep multiple ages in the same househ but with NO BARRIER

47) Do you keep other types of poultry in layer houses?

- ◯ Yes
- ◯ No

48) If yes, what type of poultry?

- ☐ Broilers
- ☐ Cocks
- ☐ Improved indigenous chicken (kienyeji)

49) About how many years or months have you been keeping layers?
Remember to put UNITS (years or months)

____

Now, I'll ask you a few questions on bird mortality and production costs. ASK THEM FOR COSTS FOR LAST PRODUCTION CYCLE OR or current production cycle if they have not completed a full cycle

50) Can you estimate the money the farm spent on medicine/vaccines/and disenfectants the last production cycle?

- ◯ Yes
- ◯ No

Could you provide an estimate of the following:

51) Number of birds that died during last production cycle or current production cycle if they have not completed a full cycle

____

52) Lump sum costs of vaccination during last production cycle or current production cycle if they have not completed a full cycle
In Kshillings. Write DK if DON"T KNOW

____

53) Lump sum costs of medicines during last production cycle
In Kshillings. Write DK if DON"T KNOW

____

54) Lump sum costs of egg boosters during last production cycle or current production cycle if they have not completed a full cycle
In Kshillings. Write DK if DON"T KNOW

____

55) Lump sum costs of feed during last production cycle or current production cycle if they have not completed a full cycle
In Kshillings. Write DK if DON"T KNOW

56) Lump sum costs of disinfectants during last production cycle or current production cycle if they have not completed a full cycle
In Kshillings. Write DK if DON"T KNOW

57) What is your USUAL starter flock?

- ◯ Day old chicks
- ◯ Grower/pullets
- ◯ Other

Specify other.

58) What is the source of your starter flock (day-old chicks/grower stock) SELECT ALL

- ☐ Directly from the Hatchery
- ☐ Agrovet
- ☐ Other Farmers
- ☐ Trader/Broker
- ☐ Other

Specify other.

59) Does your starter flock come vaccinated against some diseases?

- ◯ yes
- ◯ no
- ◯ Don't Know

60) What do you get with your starter flock package? SELECT ALL

- [ ] Chicks
- [ ] Feed
- [ ] Medicine
- [ ] Vaccines
- [ ] Multivitamins
- [ ] Liguid Paraffin
- [ ] Glucose
- [ ] Other

Specify other.

Now, I would like to ask you a few questions about the health of your poultry

61) What are the most common DISEASE SYMPTOMS your layers show? Please Select All

Symptoms listed in alphabetical order

- [ ] Abnormal eggs (small/large/round, thin/rough shell,)
- [ ] Blindness
- [ ] Bloody Droppings/Diarrhea
- [ ] circling
- [ ] Diarrhea
- [ ] Discharge from Eyes
- [ ] Drop in Egg Production
- [ ] Dullness
- [ ] Lameness
- [ ] Lesions on skin
- [ ] Paralysis
- [ ] Pecking each other
- [ ] Sleepiness
- [ ] Sneezing
- [ ] Snoring
- [ ] Sudden Death
- [ ] stargazing
- [ ] Swollen Eyes
- [ ] twisted neck
- [ ] White Diarrhea
- [ ] Wounds where the egg comes out
- [ ] Yellow/brown diarrhea
- [ ] Other

Specify other.

____

62) What are the most common diseases your layers get? Please Select All

- [ ] Coccidiosis
- [ ] Coryza
- [ ] CRD (Chronic Respiratory Disease)
- [ ] Egg Perinoitis
- [ ] Fowl Pox
- [ ] Fowl typhoid
- [ ] Gumboro Disease
- [ ] I don't know any diseases
- [ ] Infectious Bronchitis
- [ ] Marek's Disease
- [ ] My birds never get disease
- [ ] Newcastle Disease (kihuruto)
- [ ] Worms

63) Where do you mostly obtain INFORMATION/ADVICE on disease treatment for your sick layers?

- ○ Other farmers
- ○ Government vet services
- ○ Private vet services
- ○ Veterinary laboratory
- ○ Agrovet
- ○ Manufacturers
- ○ I never get advice because I know what to do

Please think about when your layers need to be treated

64) How often do these people give you advice/instructions on bird health	never	rarely	sometimes	almost always
65) Family/friends	○	○	○	○
66) NGOs	○	○	○	○
67) agrovet	○	○	○	○
68) feed or drug distributor	○	○	○	○
69) a government veterinarian	○	○	○	○
70) a private veterinarian	○	○	○	○
71) a vet laboratory	○	○	○	○

72) How often do you take samples/sick birds to a veterinarian for diagnosis?

- ◯ never/rarely
- ◯ Sometimes
- ◯ Always whenever disease strikes

73) What challenges do you face in trying to get veterinary health services? Select All

- ☐ High Cost
- ☐ Distance
- ☐ Too few services
- ☐ Lack of follow-up
- ☐ Existence of Quacks
- ☐ SLOW response time/turn around
- ☐ NO RESPONSE
- ☐ No challenges

74) What challenges do you face in trying to get diagnosis at a veterinary laboratory? Select All

- ☐ High Cost
- ☐ Distance to Lab
- ☐ Not aware of lab
- ☐ Don't know what to take to lab
- ☐ Slow Services
- ☐ No challenges

75) Are there some medicines that used to work in the past that are not as effective at treating diseases in the present?

- ◯ yes
- ◯ no
- ◯ I don't know
- ◯ I don't use medicine

76) Why do you believe the medicines are less effective?

____

77) What do you do when you observe that the medicines you used regularly on your birds are no longer effective?

- [ ] Try a different antibiotic
- [ ] Increase drug dosage
- [ ] Combine with other drugs
- [ ] Consult with animal health professional
- [ ] Shift to herbal medicine

78) Do you know what antibiotics are?

- ( ) Yes
- ( ) No

79) Select Their Answers (DO NOT LIST BUT SELECT ALL)

- [ ] medicine that PREVENTS diseases
- [ ] medicine that KILLS disease
- [ ] medicine that KILLS germs
- [ ] medicines that KILL bacteria
- [ ] medicines that PROMOTE GROWTH
- [ ] medicine increases EGG LAYING
- [ ] medicine that KILLS VIRUSES
- [ ] Other

Specify other.

____

80) Where do you USUALLY get your antibiotics from?

- [ ] agrovet
- [ ] government vet/paravet
- [ ] private vet/paravet
- [ ] a friend
- [ ] a feed distributor
- [ ] middlemen
- [ ] I've never purchased antibiotics
- [ ] Other

Specify other.

____

81) Think about when you purchase antibiotics, how does it USUALLY happen?

- I know what I need and I just tell the them the medicine name and they don't give me INTRUCTIONS ON USE
- I know what I need and I just tell the them the medicine name and they GIVE ME INTRUCTIONS ON USE
- I tell them symptoms of my birds and they tell me the antibiotic I need but NO INSTRUCTIONS ON USE
- I tell them the symptoms of my birds and they tell me the antibiotic I need and INSTRUCTIONS ON USE
- I take a sick bird to the agrovet and they tell me the antibiotics
- I never purchase antibiotics

82) When you buy antibiotics, do you usually have a prescription/or does veterinarian bring?

- never
- rarely
- sometimes
- almost always

83) When you buy antibiotics, does the SELLER require you to have a prescription?

- never
- rarely
- sometimes
- almost always

84) Why did you usually not have a presciption?

---

85) Think about YOUR LAYERS, how often are they given antibiotics in one month?

- Never
- One or Two
- 3 to 5 times
- 6 to 10 times
- Over 10 times

86) Think about ALL YOUR ANIMALS and YOUR FAMILY, how often are they given or taken antibiotics in one month?

- Never
- One or Two
- 3 to 5 times
- 6 to 10 times
- Over 10 times

87) Please list all the reasons you use antibiotics on your animals? Please Select All

- [ ] To help sick animals get better
- [ ] To PREVENT A GROUP OF ANIMALS from getting sick from other animals
- [ ] To PREVENT A SINGLE ANIMAL from getting sick
- [ ] To help animals GROW FASTER
- [ ] To help animals GROW BIGGER
- [ ] To help birds LAY MORE EGGS
- [ ] I don't use antibiotics on animals
- [ ] Other

Specify other.

Now, FOR YOUR BIRDS, please think about when give antibiotics

How often do the following people administer antibiotics?	never	rarely	sometimes	almost always
88) Farm Owner	○	○	○	○
89) Family or Friends of Farm Owners	○	○	○	○
90) Farm Manager	○	○	○	○
91) Agrovet	○	○	○	○
92) Feed Company Person	○	○	○	○
93) Extension worker	○	○	○	○
94) Govt. Vet	○	○	○	○
95) Priv. Vet	○	○	○	○
96) Vet lab worker	○	○	○	○

96) Do you give antibiotics when you get your day-old chicks?

- ○ never
- ○ rarely
- ○ sometimes
- ○ almost always

97) When a few of your birds get sick do you usually give medicine to all of them (for example, in a drinker)?

- never
- rarely
- sometimes
- almost always

98) Do you use an isolation chamber when a few of your birds are sick?

- never
- rarely
- sometimes
- almost always

99) Do you use human medicines to treat your birds?

- never
- sometimes
- almost always
- don't know

100) What medicines are they?

---

101) Do you ever give birds a LARGER DOSE than the recommended dose?

- never
- rarely
- sometimes
- almost always

102) Briefly record why they DO give a larger dose

---

103) Do you ever give birds a SMALLER DOSE than the recommended dose?

- never
- rarely
- sometimes
- almost always

104) Briefly record why they DO give a smaller dose

---

105) Do you ever STOP using medicine before the full dose because the animal has improved?

- never
- rarely
- sometimes
- almost always

106) Do you ever use expired medicines?

- never
- rarely
- sometimes
- almost always

107) Why do you use expired drugs?

---

108) If the antibiotics you have become expired, what do you USUALLY do with them?

- Will use them until they are finished
- Will not use them and bury
- Throw away in latrine
- Throw away in refuse pit
- Return them back to where you bought them
- I've never had drugs expire
- Other

Specify other.

---

109) When a bird has died, what do you USUALLY do with it?

- ◯ Consume at home
- ◯ Sell it
- ◯ Throw it in a refuse pit
- ◯ Throw it in a pit latrine
- ◯ Give it to dogs
- ◯ Bury it
- ◯ Share with neighbors and friends
- ◯ Burn it
- ◯ Other

Specify other.

____

110) Have you ever heard about "antibiotic resistance" or "antimicrobial resistance"?

- ◯ Yes
- ◯ No

111) How much do you believe antibiotic or antimicrobial resistance will impact you, your family/friends, and your animals?

- ◯ no impact
- ◯ a little impact
- ◯ a large impact
- ◯ I don't know about AMR

112) HOW do you think antibiotic/antimicrobial resistance will impact your livelihood?

- ☐ Loss of animals/money from farming
- ☐ Loss of human life (you, your family, your friends, your community)
- ☐ Other

Specify other.

____

113) Who told you about antibiotic/antimicrobial resistance?

- ☐ Friend or Neighbor
- ☐ Family Member
- ☐ Farm Manager
- ☐ Caretaker
- ☐ Poultry Drug Shop Employee
- ☐ Feed Company Person
- ☐ Extension worker
- ☐ Govt. Vet
- ☐ Priv. Vet
- ☐ Vet lab worker
- ☐ Radio
- ☐ Print Media
- ☐ I don't know about antibiotic or antimicrobial resistance
- ☐ I learned about antimicrobial resistant when I gave my chicken medicine and they did not recover

NOW, I WILL READ YOU A SET OF STATEMENTS AND ASK YOU HOW MUCH YOU AGREE WITH THEM. I'll ask whether you agree, neither agree nor disagree, or disagree

AGREE

114) How much do you agree with the statement that "You can stop giving an animal a full course of antibiotic if their symptoms are improving."

- ○ agree
- ○ I'm not sure
- ○ disagree

115) How much do you agree with the statement that "if medicines are given TOO OFTEN then they might stop working?"

- ○ agree
- ○ I'm not sure
- ○ disagree

116) How much do you agree with the statement that "giving animals that are not sick antibiotcs will PREVENT THEM FROM BECOMING SICK in the future"

- ○ agree
- ○ I'm not sure
- ○ disagree

117) How much do you agree with the statement that "giving animals antibiotcs can help them GROW BIGGER, FASTER, FATTER, boost egg production/size"

- ◯ agree
- ◯ I'm not sure
- ◯ disagree

118) How much do you agree with the statement that "it is important to get CONSULTATION FROM A VETERINARIAN before giving antibiotics to the animals?

- ◯ agree
- ◯ I'm not sure
- ◯ disagree

119) How much do you agree with the statement that "after using antibiotics on an animal, you should WAIT sometime before using the PRODUCTS from it, such as bird meat/eggs/milk"

- ◯ agree
- ◯ I'm not sure
- ◯ disagree

120) Can animals and animal products from sick animals transmit disease to humans?

- ◯ yes
- ◯ no
- ◯ Don't Know

121) Do you know what antibiotic residues are?

- ◯ Yes
- ◯ No

122) What do you USUALLY do with EGGS under CURRENT TREATMENT with antibiotics/antimicrobials or during WITHDRAWAL PERIOD?

- ☐ Consume at home
- ☐ Sell it
- ☐ Throw it in a refuse pit
- ☐ Throw it in a pit latrine
- ☐ Give it to dogs
- ☐ Bury it
- ☐ Share with neighbors and friends
- ☐ Burn it

123) Has an animal health worker/poultry drug shop/veterinarian/NGO ever told you about withdrawal periods: that is, you have to wait to sell the animal or eat a meat/egg/milk after giving the animal medicine?

- ◯ Yes
- ◯ No

Now, I'd like to ask some questions about vaccines

124) Do you know what vaccines (chanjo) are?

If they say something like "PREVENT DISEASE LONG TERM" check yes

- ◯ Yes
- ◯ No

125) What vaccines do you give to your layers? Please Select ALL

- ☐ Newcastle
- ☐ Newcastle Plus IB
- ☐ Gumboro
- ☐ Fowl typhoid
- ☐ Fowl pox
- ☐ Marek's Disease
- ☐ Don't know
- ☐ Other

Specify other.

____

126) What vaccines do you give to your OTHER BIRD (broilers/indigenous)? Please Select ALL

- ☐ Coccidiosis
- ☐ Newcastle Diseases
- ☐ Gumboro Disease
- ☐ Fowl Pox
- ☐ Fowl typhoid
- ☐ Marek's Disease
- ☐ I ONLY HAVE LAYERS NOT ANY OTHER BIRDS
- ☐ None
- ☐ Other

Specify other.

---

127) Have your layers come down with the disease they have been vaccinated for?

- ○ never
- ○ rarely
- ○ sometimes
- ○ almost always

128) Where do you purchase your vaccines from? Please Select ALL

- ☐ agrovet
- ☐ government vet
- ☐ feed distributor
- ☐ private vet
- ☐ Import from outside Kenya
- ☐ friends
- ☐ farmer associations
- ☐ Pharmaceutical company

129) Who USUALLY vaccinates the birds or layers if vaccination is GIVEN THROUGH WATER?

- ○ Myself
- ○ family/friends
- ○ government health services
- ○ private health services
- ○ Agrovet
- ○ Informal private practioners

130) Who USUALLY vaccinates the birds or layers if vaccination is given by INJECTION?

- ○ Myself
- ○ family/friends
- ○ government health services
- ○ private health services
- ○ Agrovet
- ○ Informal private practioners

131) How do you store your vaccines before use?

- [ ] Transport in cold chain and use immediately upon purchase
- [ ] Store at room temperature and use later (at least more than 2 hours after purchase)
- [ ] Store in the refridgerator

132) If you have extra vaccine (doses is higher than flock size) what do you do with i?

- [ ] share it with another farmer
- [ ] discard it
- [ ] use all of it
- [ ] store to use during the next vaccination
- [ ] I always buy exact dose

133) What challenges do you normally face with getting or administering vaccinations? Please LIST ALL CHOICES and SELECT ALL

- [ ] Unavailability
- [ ] High Cost of professional administration
- [ ] lack of information on what vaccines and when to use (don't always know where and how to get them)
- [ ] Nobody available to give vaccine
- [ ] High cost of vaccines
- [ ] Far distance to purchase vaccines
- [ ] Don't have access to proper storage/handling
- [ ] Lack of appropriate doses
- [ ] No challenges
- [ ] Other

Specify other.

____

134) How much do you agree with the statement that "using vaccines can prevent the use of antibiotics?"

- ( ) agree
- ( ) I'm not sure
- ( ) disagree

Now, I'd like to take you a few pictures of your layer houses

135) Record the number of layer houses (BUT ONLY TAKE PICTURES OF THE BIGGEST!)

____

136) How many birds are in the main layer house?

____

137) How many weeks are the birds in the main layer house?

____

138) Record housing wall type of MAIN layer house

- ☐ Iron Sheets
- ☐ Brick/Stone
- ☐ Wood/Timber
- ☐ Wire/Mesh
- ☐ Mud
- ☐ Other

Specify other.

____

139) Record housing floor type of MAIN layer house

- ○ Earthen
- ○ Cement/Concrete
- ○ Wooden block (non-slated)
- ○ Wooden slated floor
- ○ Wire
- ○ A combination of wood and wire mesh
- ○ Other

Specify other.

____

140) Record type of litter used in Main layer house

- ◯ Straw(Grass/Wheat)
- ◯ wood shavings
- ◯ sand
- ◯ rice husks
- ◯ used paper
- ◯ none
- ◯ Other

Specify other.

---

141) Do you treat your litter before putting it on the floor of your layer house?

- ◯ never
- ◯ rarely
- ◯ sometimes
- ◯ almost always

142) What do you USUALLY use to treat your litter? Select All that Apply

- ☐ disinfectant
- ☐ Sun Drying
- ☐ Other

Specify other.

---

143) What do you do with the used litter? Please Select All

- ☐ Use as Livestock Feed (cattle)
- ☐ Use as Livestock Bedding
- ☐ Put into the fields compost/manure
- ☐ Sell to neighbors
- ☐ re-use
- ☐ burn
- ☐ Bury it
- ☐ Throw it away
- ☐ Other

Specify other.

____

144) Observe and Record drinker type (the majority of drinkers)

- ◯ Commercial plastic drinkers
- ◯ Commercial metal drinkers
- ◯ Improvised (plastic)

145) Observe and record drinker positioning

- ◯ Automatic drinkers
- ◯ Nipple drinkers
- ◯ Drinkers hanged above the floor
- ◯ Drinkers placed on the floor

146) How many drinkers do you currently use in MAIN layer house?

____

147) Record drinker material

- ◯ Plastic
- ◯ Metal
- ◯ Wood
- ◯ Clay
- ◯ Other

Specify other.

____

148) Where do you USUALLY get your water for your layers? Ask about source if not treated

- ◯ borehole
- ◯ tap water
- ◯ rain water
- ◯ river water
- ◯ hand dug well
- ◯ Water vendors
- ◯ Other

Specify other.

____

149) How often do you USUALLY clean the drinkers?

- ◯ Never Clean
- ◯ Daily
- ◯ Once a week
- ◯ Twice a week
- ◯ Once or twice a month
- ◯ Only after the production cycle

150) What do you USUALLY clean your drinkers with?

- ◯ Water Only
- ◯ Water and Soap Only
- ◯ Water and disinfectant Only
- ◯ Water, disinfectant, and Soap

151) Observe and Record if areas around drinkers are moist

- ◯ very damp
- ◯ somehow damp
- ◯ dry

152) Is the availability or quality of water a source of problem in raising your bird?

- ◯ Yes
- ◯ No

153) Do you USUALLY treat the water before putting into drinker?

- ◯ No, I do not USUALLY treat
- ◯ Yes, I treat with chemicals (disenfectant, chlorine)
- ◯ Yes, I boil

154) How many feeders are in main layer house?

____

155) Record feeder type

- ◯ Plastic
- ◯ Metal
- ◯ Wooden
- ◯ Rubber (tire)
- ◯ Oyster Shells

156) How often do you USUALLY clean the feeders?

- ◯ Never Clean
- ◯ Daily
- ◯ Once or twice a week
- ◯ Once a month
- ◯ Only after cycle ends

157) What do you USUALLY clean your feeders with?

- ◯ Water
- ◯ Water and Soap Only
- ◯ Water and disinfectant
- ◯ Water, disinfectant, and Soap

158) What feed distributor do you use? Please Select All

- ☐ Own made feeds: I mix it myself
- ☐ I bring a local company the ingredients and they mix
- ☐ Shop / kiosk
- ☐ Directly from a manufacturing company
- ☐ Distributor
- ☐ Agrovet

159) Take photo of area where they store feed

Click here to upload file. (< 10MB)

160) Observe and Record how damp the area is. Is the storage area damp?

- ◯ very damp
- ◯ somehow damp
- ◯ dry

161) Record the quality of ventilation in layer house

Or should we ask only artificial/manual

- ☐ Two walls open along the length of the house not the width
- ☐ 50 cm height of wall
- ☐ Gap in roof
- ☐ Structure is more than 10 meters away from other structures

162) Do you have a tire bath at MAIN entrance?

- ◯ Yes
- ◯ No

163) Do you have a footbath at every entrance to LAYER HOUSE?

- ◯ Yes
- ◯ No, I do have footbaths but not at every house
- ◯ No, I don't have ANY FOOTBATHS

164) Record the type of footbath

- ◯ concrete trough with water
- ◯ concrete trough with disenfectant
- ◯ mat soaked with disenfectant
- ◯ mat soaked with water
- ◯ wood shavings soaked with disenfectant
- ◯ wood shavings soaked with water
- ◯ foot dip with water
- ◯ foot dip with disenfectant

165) How often do you change the footbath contents?

- ◯ Every day
- ◯ Once or twice per week
- ◯ Once a month
- ◯ Never change (just fulfills when it dries up)

166) Observe and Record if thermometer present in layer house

- ◯ Yes
- ◯ No

Protective Gear

**Do you own any of these protective gear?**	I have	I use	I have for different houses
**167) Protective boots**	☐	☐	☐
**168) Masks**	☐	☐	☐
**169) Goggles**	☐	☐	☐
**170) Gloves**	☐	☐	☐

170) How often do you clean the protective gear?

- ◯ after everytime entering the house
- ◯ after a few times in the poultry house
- ◯ only when dirty
- ◯ I don't have any protective gear

171) Do people wear protective boots/slippers when they go into the layer house to get eggs, feed/give water OR ANY OTHER REASON?

- ◯ after everytime entering the house
- ◯ after a few times in the poultry house
- ◯ only when dirty
- ◯ I don't have any protective gear

172) Do you USUALLY clean and disinfect housing and equipment after selling a batch?

- ◯ No
- ◯ Yes
- ◯ Not applicable, first batch

173) How long do you USUALLY let the layer house rest before taking in the new batch?

- ◯ no rest
- ◯ less than one week
- ◯ between 1 and 2 weeks
- ◯ about 2 weeks
- ◯ Over a month
- ◯ Not applicable, first batch

174) Do family/friends/customers come and walk inside the layer house?

- ◯ never
- ◯ rarely
- ◯ sometimes
- ◯ almost always

175) Is it common for other animals to go into layer house?

- ◯ never
- ◯ rarely
- ◯ sometimes
- ◯ almost always

FINALLY, A FEW LAST QUESTIONS

176) Does anyone at the farm have any training related to animal health?

- ◯ Yes
- ◯ No

177) What did the trainings cover? Please Select ALL

- ☐ record-keeping financial management marketing
- ☐ production
- ☐ animal health
- ☐ Other

Specify other.

---

178) Do you take farm records? If yes, please list what types. PLEASE SELECT ALL

- ☐ I don't keep any records
- ☐ Mortality
- ☐ Feed Records
- ☐ Medicine used
- ☐ General Financial Records
- ☐ Other

Specify other.

---

179) Outreach

180) Can I have your phone number?

---

181) Do you use Whatsapp?

- ◯ Yes
- ◯ No

182) Is it the same number you gave previous?

- ◯ Yes
- ◯ No

183) Can I have your WhatsApp number

---

184) Where do you get general news. That is news about weather, government, etc.

- ◯ Radio
- ◯ Internet pages
- ◯ Television programs
- ◯ Print Newspapers
- ◯ Fliers / paper handouts
- ◯ People you know

185) Select all animal houses that are within 10 meters of the layer house

- [ ] Broilers
- [ ] Cattle
- [ ] Ducks
- [ ] Geese
- [ ] Goats
- [ ] Guinea fowl
- [ ] Human House
- [ ] Indigenous chickens
- [ ] No other animals/animal house
- [ ] Pig
- [ ] Rabbits
- [ ] Sheep
- [ ] Turkeys

Now, ASK THE RESPONDENT TO GO GET THE MEDICINE THEY USE

186) Take photo of frontal view of the MAIN layer house

Click here to upload file. (< 10MB)

187) Record GPS of house

latitude (x.y °)

____

longitude (x.y °)

____

altitude (m)

____

accuracy (m)

____

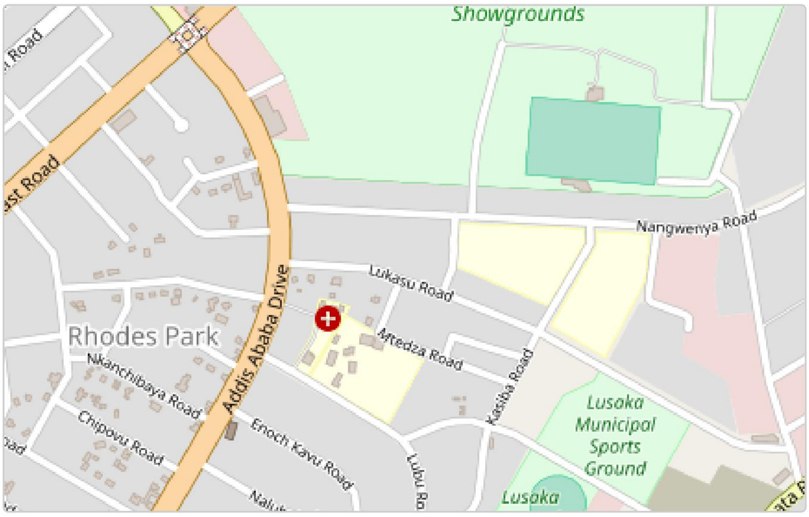

Show them the posters and ask what medicines they normally use

- [ ] 100 grams Bimeda
- [ ] Aliseryl Bottle
- [ ] Aliseryl Sachet
- [ ] AMNIN'TOTAL SACHET
- [ ] AMNIN'TOTAL BOTTLE
- [ ] Amoxicilina 500 mg/g
- [ ] AMOXY-COL WSP SACHET
- [ ] AMPROCOX BOTTLE
- [ ] AMPROCOX SACHET
- [ ] Amprolium 250 WSP
- [ ] Amprolium 20%
- [ ] Antibact
- [ ] Ashoxy 20% Sachet
- [ ] Ashoxy egg
- [ ] Biospark-Y
- [ ] Colistina 3000
- [ ] Colistine-Sulphate Soluble Powder Sachet
- [ ] CRD Stop Sachet
- [ ] CRD Powder
- [ ] Diamond MVS Broiler Boost
- [ ] Doxin
- [ ] Egocin Powder
- [ ] Enrocoli 10% Oral Solution
- [ ] Intertrim 400
- [ ] Layers Extra Sachet
- [ ] Levasol 200 WSP Sachet
- [ ] Magic Boost mayai
- [ ] Neo-oxy egg formula
- [ ] Neo-oxy vitamin wsp bottle
- [ ] Neo-oxy vitamin wsp sachet
- [ ] Nemovit Bottle
- [ ] Nemovit Sachet

- ☐ Oxysol Plus
- ☐ Oxytetracyline 50% soluble powder
- ☐ Piperazine Sachet
- ☐ Super Egg Formula Plus
- ☐ Tetraoxy Chick Formula Sachet
- ☐ Tetracolivit Sachet
- ☐ Tylo 200 WSP
- ☐ Tylodox Extra WSP
- ☐ Tylosin 75%
- ☐ WS (Multivits)
- ☐ WS (antibiotics)
- ☐ Venlyte Powder sacthet
- ☐ Vetoxy 20
- ☐ Vetpro Magic Boost
- ☐ Vigal 2X Sachet
- ☐ Vitamit-Egg Extra

188) We would like to collect fecal samples at a later date. We would collect fecal samples from poultry, swabs, and water. We will follow best protocol for entering the poultry house. Would it be okay if we came back?

- ○ Yes
- ○ No

closing

Tell the informant that you appreciate their time and that their responses will help us succeed in our project.

Also tell them that after analyzing the results we will come and talk to them about what we found and get their inout on interventions to improve health of their birds.

REMEMBER to write their last name and ID on next page. For example Caudell_100
