# Supplementary material for: Understanding Antimicrobial Use Contexts in the Poultry Sector: Challenges for Small-Scale Layer Farms in Kenya
Source: Antibiotics (Basel). 2021 Jan 22;10(2):106. doi: 10.3390/antibiotics10020106 (PMC7911778; doi:10.3390/antibiotics10020106)
Supplement: Supplementary file 1 [file antibiotics-10-00106-s001.zip › antibiotics-1061982-supplementary/Supplement C.docx]

Supplement C: Additional study details


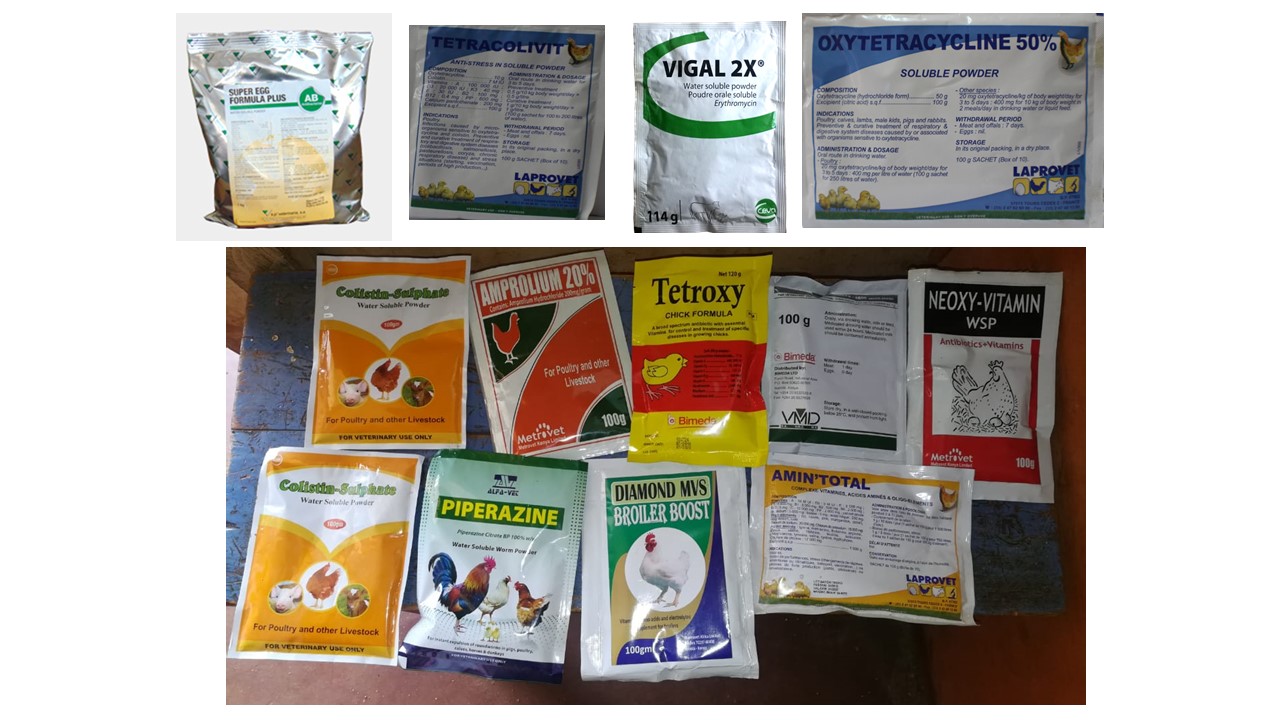


**Figure S1.** Example of a poster used to collect antimicrobial use data.

**Table S1.** Demographic Results.

| **Variable** | **N = 76** | **%** |  |
| --- | --- | --- | --- |
| **Sex** |  |  |  |
| Female | 40 | 53 |  |
| Male | 36 | 47 |  |
| **Age group** |  |  |  |
| 20-24 | 4 | 5 |  |
| 25-29 | 5 | 7 |  |
| 30-34 | 5 | 7 |  |
| 35-39 | 6 | 8 |  |
| 40-44 | 9 | 12 |  |
| 45-50 | 14 | 18 |  |
| >50 | 33 | 44 |  |
| **Main occupation of the owner** |  |  |  |
| Farmer | 52 | 68 |  |
| Formal employment | 3 | 4 |  |
| Informal employment | 7 | 9 |  |
| Retired | 14 | 18 |  |
| **Education level of the owner** |  |  |  |
| Completed primary | 22 | 29 |  |
| Completed secondary | 24 | 32 |  |
| Completed tertiary | 19 | 25 |  |
| Uncompleted primary | 5 | 7 |  |
| Uncompleted secondary | 5 | 7 |  |
| Uncompleted tertiary | 1 | 1 |  |
| **Languages farm owner can read** |  |  |  |
| English, Kiswahili, Kikuyu | 64 | 84 |  |
| Kiswahili, Kikuyu | 12 | 16 |  |

**Table S2.** Farm characteristics .

| **Variable** | **mean** | **sd** | **min** | **max** |
| --- | --- | --- | --- | --- |
| Number of layers | 1080 | 2293 | 90.00 | 19000.00 |
| Number of layer houses at the farm | 2.39 | 1.68 | 1.00 | 11.00 |
| Number of years farmer has kept layers? | 10.88 | 8.39 | 0.25 | 38.00 |
| Number of broilers at farm | 5.54 | 16.00 | 0.00 | 100.00 |
| Number of dairy cattle at farm | 2.79 | 2.99 | 0.00 | 16 |
| Number of beef cattle at farm | 0.39 | 0.98 | 0.00 | 7.00 |
| Number of sheep at farm | 0.66 | 1.33 | 0.00 | 5.00 |
| Number of goats at farm | 0.83 | 1.64 | 0.00 | 8.00 |
| Number of pigs at farm | 0.91 | 3.46 | 0.00 | 22.00 |
| Number of dogs at farm | 1.13 | 1.21 | 0.00 | 5.00 |
| Number of rabbits at farm | 0.24 | 0.88 | 0.00 | 4.00 |
| Number of geese at farm | 0.12 | 0.83 | 0.00 | 7.00 |
| Number of ducks at farm | 0.30 | 1.05 | 0.00 | 7.00 |
| Number of turkeys at farm | 0.03 | 0.23 | 0.00 | 2.00 |

**Table S3.** Common Symptoms reported in layers in Gatundu North.

| **Scheme 92.** | **% HHs**  **reporting** |
| --- | --- |
| Abnormal eggs | 92 |
| Drop in egg production | 87 |
| Snoring | 72 |
| Bloody droppings diarrhea | 70 |
| Yellow brown diarrhea | 70 |
| White diarrhea | 59 |
| Twisted neck | 47 |
| Dullness | 46 |
| Sudden death | 46 |
| Sleepiness | 43 |
| Sneezing | 43 |
| Swollen eyes | 42 |
| Diarrhea | 38 |
| Blindness | 33 |
| Cloaca wounds | 33 |
| Lameness | 22 |
| Discharge from eyes | 20 |
| Circling | 16 |
| Star gazing | 14 |
| Lesions on skin | 12 |
| Paralysis | 8 |
| N | 76 |
